# Genome-Wide Analysis of the RNA Helicase Gene Family in *Gossypium raimondii*

**DOI:** 10.3390/ijms15034635

**Published:** 2014-03-17

**Authors:** Jie Chen, Yujuan Zhang, Jubo Liu, Minxuan Xia, Wei Wang, Fafu Shen

**Affiliations:** State Key Laboratory of Crop Biology, College of Agronomy, Shandong Agricultural University, Tai’an 271018, Shandong, China; E-Mails: chenj_19@163.com (J.C.); Zhangj_19@163.com (Y.Z.); liujubo2014@163.com (J.L.); Xiaj_19@163.com (M.X.); sdauww@126.com (W.W.)

**Keywords:** cotton, RNA helicase, gene expansion, structure, fiber

## Abstract

The RNA helicases, which help to unwind stable RNA duplexes, and have important roles in RNA metabolism, belong to a class of motor proteins that play important roles in plant development and responses to stress. Although this family of genes has been the subject of systematic investigation in *Arabidopsis*, rice, and tomato, it has not yet been characterized in cotton. In this study, we identified 161 putative RNA helicase genes in the genome of the diploid cotton species *Gossypium raimondii*. We classified these genes into three subfamilies, based on the presence of either a DEAD-box (51 genes), DEAH-box (52 genes), or DExD/H-box (58 genes) in their coding regions. Chromosome location analysis showed that the genes that encode RNA helicases are distributed across all 13 chromosomes of *G. raimondii*. Syntenic analysis revealed that 62 of the 161 *G. raimondii* helicase genes (38.5%) are within the identified syntenic blocks. Sixty-six (40.99%) helicase genes from *G. raimondii* have one or several putative orthologs in tomato. Additionally, GrDEADs have more conserved gene structures and more simple domains than GrDEAHs and GrDExD/Hs. Transcriptome sequencing data demonstrated that many of these helicases, especially *GrDEADs*, are highly expressed at the fiber initiation stage and in mature leaves. To our knowledge, this is the first report of a genome-wide analysis of the RNA helicase gene family in cotton.

## 1. Introduction

RNA helicases are found in many organisms and have important roles in RNA metabolism. These enzymes participate in many metabolic pathways that involve the separation of double-stranded nucleic acid into single strands and the removal of nucleic-acid-associated proteins [1]. Usually, they translocate along the bound strand unidirectionally and their specific polarity depends upon the bound strand. RNA helicases potentially regulate cellular growth and differentiation, as well as responses to abiotic stress, by affecting nuclear mRNA export, translation initiation, mRNA decay, rRNA processing, cell cycle progression, and the initiation of transcription [2–4].

Based on variations within the DEAD (Asp-Glu-Ala-Asp) motif, the RNA helicase superfamily proteins can be classified into three subfamilies, which are defined by the presence of either the DEAD-box, DEAH-box, or DExD/H-box [5,6]. Multiple members of each subfamily have been found in several plants. Many of them, especially *DEAD-box* genes, are key regulators of developmental processes and responses to diverse abiotic stresses, such as salt stress, oxygen levels, light, or temperature [3]. In *Arabidopsis*, the DEAD-box RNA helicase LOS4 controls responses to low temperature and processes such as flowering and vernalization [7,8]. STRS1 and STRS2 mediate stress responses to various abiotic stresses [9]. RCF1 maintains proper splicing of pre-mRNAs and regulates responses to changes in temperature [10]. Tobacco VDL (for variegated and distorted leaf), a plastid DEAD-box RNA helicase, is essential for chloroplast differentiation and plant morphogenesis [11]. Maize DRH1 interacts with the nucleolar protein fibrillarin, MA16, and controls the metabolism of ribosomal RNA [12]. Rice ABP (ATP-binding protein) is upregulated in response to multiple abiotic stresses [13]. We reported that the DEAD-box RNA helicase AvDH1 (*Apocynum venetum* ATP-dependent DEAD-box helicase) participates in the regulation of salt tolerance in the halophyte *Apocynum venetum* [14]. The nucleolar DExD/H box RNA helicase AtMTR4 (mRNA transport protein) is required for normal rRNA biogenesis and development in *Arabidopsis* [15]. ISE2, a DEVH-box RNA helicase, has been shown to be involved in plasmodesmata function during embryogenesis in *Arabidopsis* [16]. The DEVH-box RNA helicase AtHELPS is important for responses to and tolerance of K^+^ deprivation in *Arabidopsis* [17]. The *Arabidopsis* DEAH-Box RNA helicase RID1 controls pre-mRNA splicing [18].

The recent availability of genome sequences enabled systematic investigation of the families of RNA helicase genes from *Arabidopsis*, rice, tomato, maize, and soybean. A total of 32 DEAD-box RNA helicases have been identified in *Arabidopsis* [19]; 113 and 115 RNA helicase genes have been found in *Arabidopsis* and rice, respectively [1]. Xu *et al*. [5] described the complete analysis and classification of 157 RNA helicase genes in tomato. Soon after, they reported a comparative genome-wide analysis of the RNA helicase gene families in *Arabidopsis*, rice, maize, and soybean [6]. The RNA helicase genes from *Arabidopsis*, rice, maize, and soybean were classified into three subfamilies, with the following respective numbers of genes for each subfamily: DEAD-box (50, 51, 57, and 87 genes), DEAH-box (40, 33, 31, and 48 genes), and DExD/H-box (71, 65, 50, and 78 genes) [6].

Cotton is an economically important crop grown worldwide as a source of fiber and edible oil [20,21]. RNA helicases are ubiquitous proteins that are believed to play important roles in cotton development and stress tolerance. However, only a few RNA helicases have been characterized in cotton [22,23]. *Gossypium raimondii* is a diploid species of cotton. Release of the *G. raimondii* genome enabled us to identify and analyze the family of RNA helicase genes in this species. Presently, there are a few reports on studies of cotton transcriptomics [22,24]. Here, we looked at expression of the different RNA helicases in transcriptome data obtained at Peking University (Beijing, China) and accessed through NCBI [25,26]. The present study identified 161 RNA helicase genes from the *G. raimondii* genome. These were classified into three subfamilies, which were defined by the presence of either a DEAD-box, DEAH-box, or DExD/H-box. Detailed information about the chromosomal locations, expansion, genomic structures, and phylogenetic relationships of the RNA helicase genes is provided. Transcript profiles of 161 RNA helicase genes in mature leaves and at the 0-day-post-anthesis (DPA) and 3-DPA ovule developmental stages were investigated using transcriptome-sequencing data. Our results show that most of these helicase proteins, especially GrDEADs, might function during the fiber initiation stage. This is the first report of a genome-wide analysis of the RNA helicase gene family of *G. raimondii.*

## 2. Results

### 2.1. Identification of RNA Helicase Family Genes in G. raimondii

RNA helicase usually contains a highly conserved adenosine triphosphate (ATP)-binding domain and a classical *C*-terminal domain [27–29]. To identify all members of the RNA helicase gene family in *G. raimondii*, all known *Arabidopsis* helicase gene sequences and all identified tomato RNA helicase gene sequences were used to query the protein database of *G. raimondii* using BLAST (basic local alignment search tool) analysis. This identified 161 genes, of which 151 genes had apparent helicase domains with the remaining 10 genes having either incomplete or no helicase domains. Given that the annotation of the *G. raimondii* genome indicated that these 10 genes encode helicase proteins, they were subjected to further analysis. This enabled us to classify all 161 putative helicase genes into three subfamilies based on their phylogenetic relationships and the structural features of the motif II region. The three subfamilies were defined by the presence of the DEAD-box (51 genes), DEAH-box (52 genes), and DExD/H-box (58 genes) motifs (Table 1, File S2). Based on their subfamily and the order in which they are found on chromosomes 1 through 13, the genes were renamed from *GrDEAD1* through *GrDEAD51*, from *GrDEAH1* through *GrDEAH52*, and from *GrDExD/H1* through *GrDExD/H58* (Table 1). The lengths, molecular weights, isoelectric points, and predicted subcellular localizations of each *Gossypium raimondii* helicase protein are summarized in Table 1. We found that *GrDEAD* genes were distinct from *GrDEAH* and *GrDExD/H* genes. Whereas the average GrDEAD protein contains 609 amino acids (aa), GrDEAH and GrDExD/H proteins each average approximately 1100 aa in length. The average theoretical isoelectric point (pI) of GrDEAD proteins, which is approximately 8.21, is higher than that for members of the two other families. Whereas, 74.5% of the helicases proteins analyzed were predicted to be located in the nucleus, 16.1% were predicted to reside in the cytoplasm, and others were mainly predicted to be located in the chloroplast and mitochondrion (Table 1).

### 2.2. Phylogenetic Analysis of the RNA Helicase Family Genes in G. raimondii

**Figure 1.**
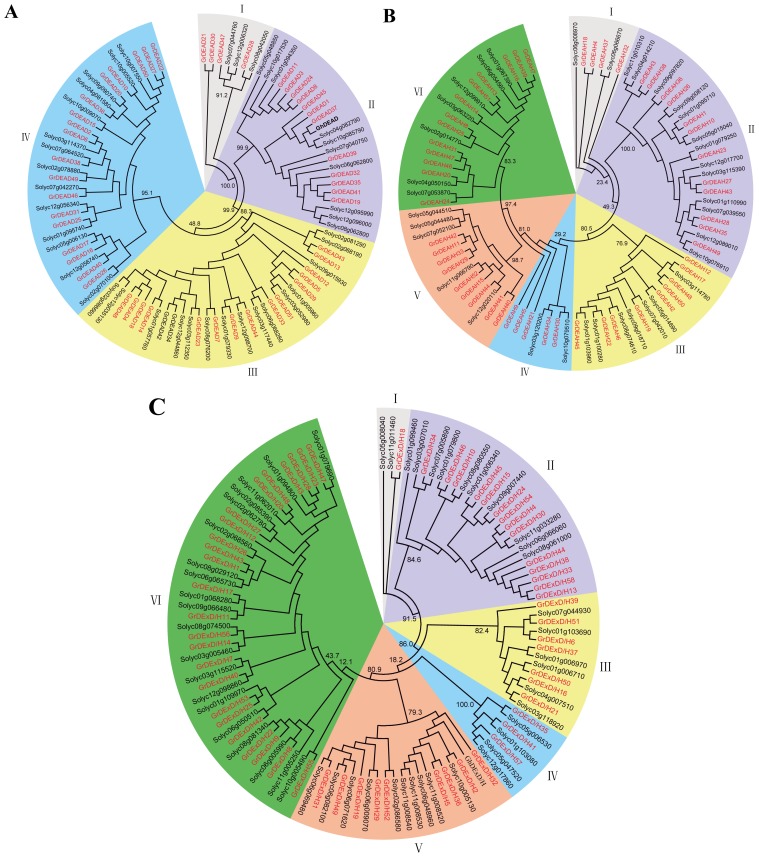
Phylogenetic analysis of RNA helicases in tomato and *G. raimondii*. (**A**) The DEAD-box RNA helicase proteins; (**B**) The DEAH-box RNA helicase proteins; (**C**) The DExD/H-box RNA helicase proteins. The amino acid sequences of the RNA helicase proteins were aligned with MUSCLE (v3.8.31) [30], and the phylogenetic tree was constructed using the maximum-likelihood (ML) method.

The phylogenetic tree of each subfamily in our study showed that the DEAD-box (Figure 1A), DEAH-box (Figure 1B) and DExD/H-box (Figure 1C) subfamilies could be further classified into four, six, and six subgroups, respectively. However, the available phylogenetic analysis of RNA helicases from *Arabidopsis*, rice, maize, and soybean [6] places these subfamilies into many more subclades. That study classified the DEAD-box, DEAH-box and DExD/H-box RNA helicase proteins from tomato into three, three, or five large subgroups, respectively. The diversity of these subclades indicates the extent of the variation of RNA helicase genes in plants.

### 2.3. Chromosomal Position and Gene Duplications

**Figure 2.**
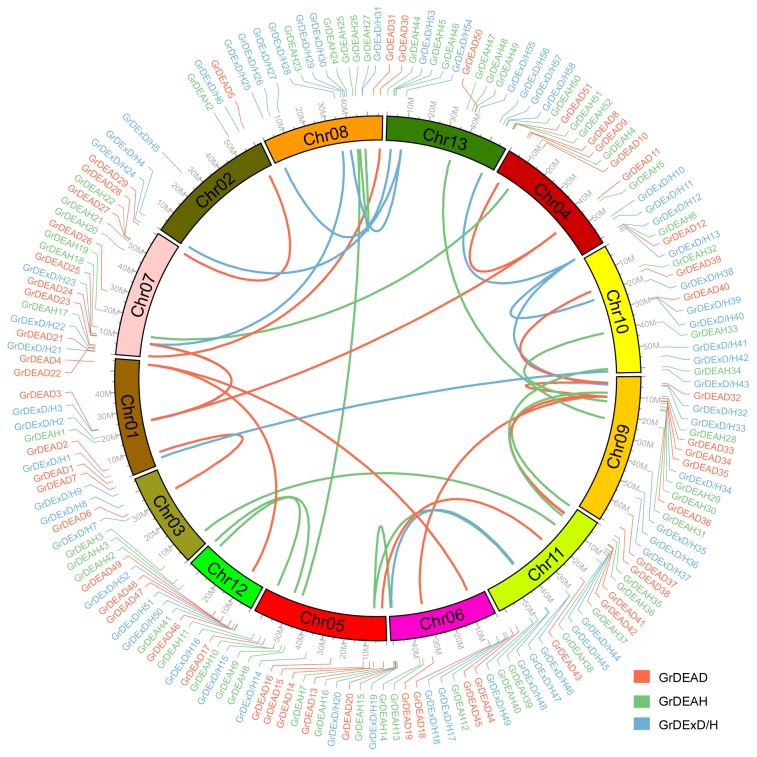
Syntenic relationships among RNA helicases in *G. raimondii*. The *G. raimondii* RNA helicases genes are shown in the outer circle, with DEAD-box, DEAH-box and DExD/H-box RNA helicase genes indicated in red, green, and blue, respectively. Genome-wide duplicated genes in the DEAD-box, DEAH-box, and DExD/H-box subfamilies are connected by red, green, and blue lines, respectively.

The 161 RNA helicase genes of *G. raimondii* are distributed across all 13 chromosomes, with different densities of their distribution along different chromosomes (Figure 2). For example, whereas chromosome 7 contained 19 RNA helicase genes, only five RNA helicase genes were annotated on chromosome 2. Based on the definition of gene clusters, a chromosomal region containing two or more genes within 200 kb can be considered to be a gene cluster [31,32]. In *G. raimondii*, 18 helicase genes were identified in nine clusters (*GrDEAD34–35*, *GrDExD/H46–47*, *GrDEAD40-GrDExD/H39*, *GrDEAH10-GrDExD/H15*, *GrDEAH6-GrDExD/H12*, *GrDEAD27-GrDEAH22*, *GrDEAH27-GrDExD/H31*, *GrDEAD48-GrDExD/H52*, and *GrDEAD47-GrDExD/H5*) that were dispersed across several chromosomes. Two pairs of genes (*GrDEAD40-GrDExD/H39* and *GrDEAH10-GrDExD/H15*) were arranged in tandem repeats on chromosomes 5 and 10, respectively. In addition, recent studies have shown that *G. raimondii* has undergone the hexaploidization event (γ-WGD) shared by the eudicots and a cotton-specific whole genome duplication [25]. To analyze the relationship between the RNA helicase genes and genome-wide duplications, we mapped the *G. raimondii* helicase genes to the duplicated blocks. We found that 62 of the 161 helicase genes (38.5%) had syntenic relationships (Figure 2, File S1). Of these, 40 genes involved only two chromosome regions, nine (*GrDEAD4-18-48*, *GrDExD/H4-30-54*, and *GrDEAH7-16-39*) spanned three chromosome regions, eight (*GrDEAD3-8-11-24* and *GrDExD/H13-33-38-58*) traversed four chromosome regions, and five (*GrDEAD19-32-35-39-41*) crossed five chromosome regions (Figure 2, File S1).

**Figure 3.**
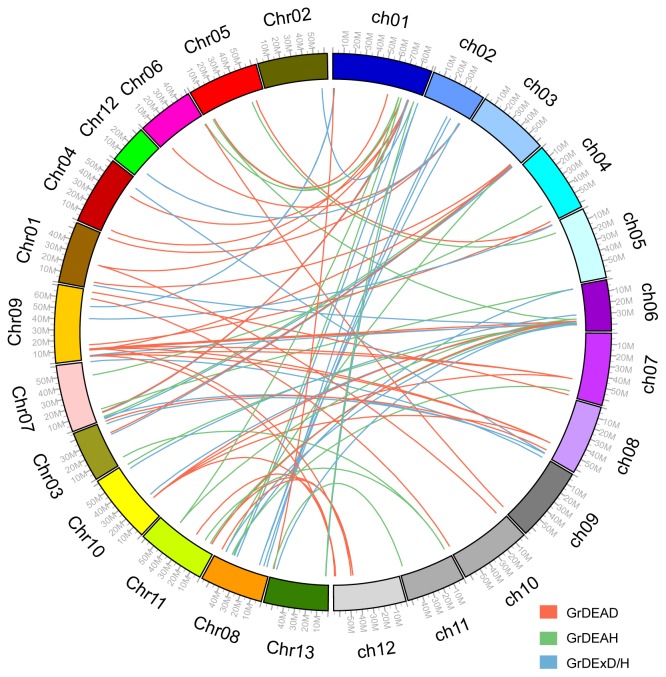
Syntenic relationships between RNA helicases of tomato and *G. raimondii. ch*, chromosome of tomato; *Chr*, chromosome of *G. raimondii*. DEAD-box, DEAH-box, and DExD/H-box genes are indicated in red, green, and blue, respectively. The putative orthologous RNA helicase genes belonging to the DEAD-box, DEAH-box, and DExD/H-box subfamilies are connected by red, green, and blue lines, respectively.

We also examined the orthologous relationships between helicase genes from *Gossypium raimondii* and tomato, given that orthologs often retain equivalent functions during the course of evolution [33]. We found that 66 (40.99%) helicase genes from *Gossypium raimondii* have one or several putative orthologs in tomato (Figure 3, File S1). Of these, 27, 16, and 23 were assigned to the DEAD, DEAH, and DExD/H-box subfamilies, respectively (File S1). One member in DEAD-box family, GrDEAD7, is an ortholog of SIDEAD27 in tomato and STRS1 in *Arabidopsis*. Whereas, GrDEAD37 is an ortholog of SIDEAD34 in tomato and LOS4 in *Arabidopsis*, GrDExD/H35 is an ortholog of SIDExD/H21 in tomato and ISE2 in *Arabidopsis* (File S1).

### 2.4. Structures and Domain Analysis of the Putative Helicase Genes

**Figure 4.**
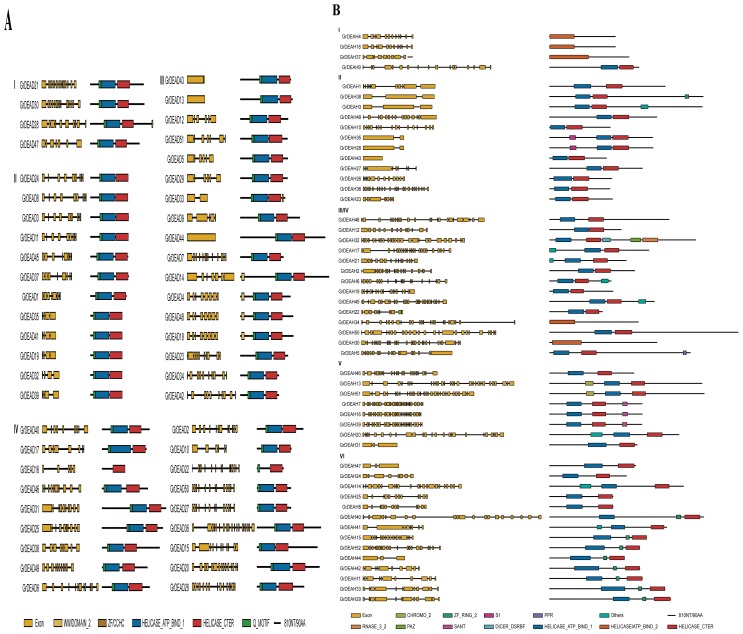

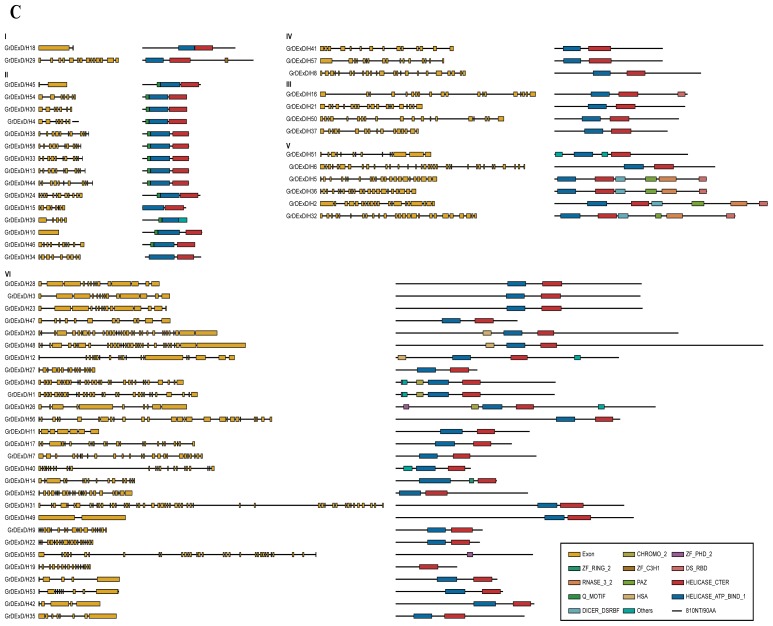
Gene structure and domain analysis of RNA helicases in *G. raimondii*. (**A**) The DEAD-box RNA helicase genes; (**B**) The DEAH-box RNA helicase genes; (**C**) The DExD/H-box RNA helicase genes. Exons and introns of each subgroup are shown in the yellow boxes on the left. Conserved domains annotated by PROSITE are shown in colored boxes on the right.

Figure 4 shows the exon-intron structures and conserved domains of putative RNA helicase genes in each subfamily. The number and location of introns varied among subfamilies. In general, compared with the two other subfamilies, DEAD family genes had simpler structures and more conserved structural patterns than members of the two other subfamilies. The relative levels of conservation are exemplified by comparison of the high level of conservation evident in the comparisons GrDEAD21–30, GrDEAD25–31, GrDEAD27–50, GrDEAD4-18-48, GrDEAD23-34-42, GrDEAD1-37-45, GrDEAD5-12-29-51, GrDEAD3-8-11-24, and GrDEAD19-32-35-39-41 (Figure 4A), with the lower level of conservation amongst members of the GrDEAH-box and GrDExD/H-box helicase family genes evident in the comparisons GrDEAH4-18, GrDEAH38-3, GrDEAH35-28, GrDEAH25-8, GrDEAH33-29, and GrDEAH7-16-39 (Figure 4B), as well as GrDExD/H5–36, GrDExD/H43-1, GrDExD/H20–48, GrDExD/H54-30-4, GrDExD/H28-3-23, and GrDExD/H38-58-33-13-44 (Figure 4C). Gene structures within the same subgroup of all three subfamilies were also very diverse. In addition, we found that whereas genes duplicated in different parts of the genome (such as combinations *GrDEAD35-41-19* and *GrDEAD4-48-18*) had the same or similar gene structures, genes that had been duplicated in tandem (*GrDEAD40-GrDExD/H39* and *GrDEAH10-GrDExD/H15*) had different gene structures, especially in terms of the numbers of exons. Domain analysis indicated the presence of a highly conserved ATP-binding domain and a classical *C*-terminal domain in almost all of the predicted RNA helicases. Additionally, we found a Q motif in all members of the DEAD family, except for GrDEAD16. A WW domain was observed in three members of the DEAD family. We also found that many of the DEAH and DExD/H family genes were surrounded by defined folds, such as the zf-RING, dsRBDs, and HSA domains.

### 2.5. Expression of RNA Helicase Family Genes in G. raimondii

**Figure 5.**
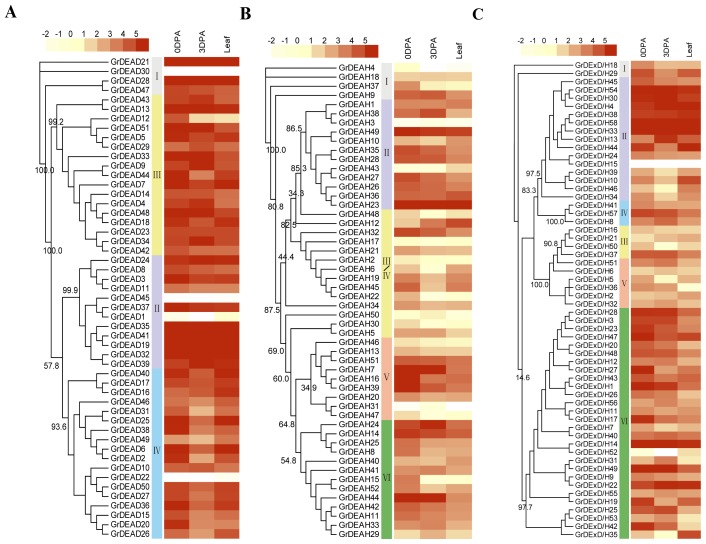
Expression patterns of RNA helicases in *Gossypium raimondii*. (**A**) The DEAD-box RNA helicase proteins; (**B**) The DEAH-box RNA helicase protein; (**C**) The DExD/H-box RNA helicase proteins. Heatmap showing the clustering of RNA helicase genes across three tissues (leaf, ovules at 0 DPA, and ovules at 3 DPA; mentioned at the top of each lane). RNA seq data (series accession number SRA048621) was obtained from the National Center for Biotechnology Information (NCBI) Sequence Read Archive (SRA) database [26]. The color scale at the top of the dendrogram indicates the relative expression levels. Color scale represents reads per kilobase per million normalized log2 transformed counts where light green indicates low level and red indicates high level.

The full-length RNA helicase protein sequences in *G. raimondii* were aligned by MUSCLE (v3.8.31) [30] and analyzed using maximum likelihood (ML) method. The attribution of proteins in Figure 5 is according to the attribution of proteins in Figure 1. Bootstrap values were calculated. The abundances of the transcripts that encode selected RNA helicases was examined at the fiber initiation stage and in mature leaves. The expression patterns of 161 RNA helicase family genes are shown in Figure 5. Of the 161 predicted genes, only six genes (those that encode GrDEAD1, GrDEAD22, GrDEAD30, GrDEAD45, GrDEAH3, and GrDExD/H15) were not expressed, whereas 141 genes were expressed both in ovules and leaves. The GrDEAD genes (Figure 5A) showed more homogenous levels of expression and an overall higher level of expression both in ovules and leaves when compared with *GrDEAH* genes (Figure 5B) and *GrDExD/H* genes (Figure 5C). Seven *GrDEAD* genes (*GrDEAD13*, *GrDEAD19*, *GrDEAD21*, *GrDEAD28*, *GrDEAD32*, *GrDEAD35*, and *GrDEAD41*), one *GrDEAH* gene (*GrDEAH23*), and three *GrDExD/H* genes (*GrDExD/H33*, *GrDExD/H54*, and *GrDExD/H58*) were expressed at high levels in all three samples. A member of the DEAD-box family, *GrDEAD7*, which is an ortholog of *Arabidopsis STRS1* was highly expressed in 0-DPA ovules and mature leaves. More than half of the genes were mainly expressed in one of the development stages and tissues tested, with 50 genes (8 *GrDEADs*, 24 *GrDEAHs*, and 18 *GrDExD/Hs*) being the most abundant in 0-DPA ovules, 25 (7 *GrDEADs*, 5 *GrDEAHs*, and 13 *GrDExD/Hs*) being the most abundant in 3-DPA ovules, and 14 (3 *GrDEADs*, 6 *GrDEAHs*, and 5 *GrDExD/Hs*) being the most abundant in mature leaves. For example, *GrDEAD37* (an ortholog of *Arabidopsis LOS4*) was highly expressed in 0-DPA ovules, whereas, *GrDExD/H35* (an ortholog of *Arabidopsis ISE2*) was expressed in mature leaves. Whereas, the similarity of the abundance profiles of transcripts encoded by duplicated genes, such as *GrDEAD19*, *GrDEAD35*, and *GrDEAD41*, suggested functional redundancy between these family members, the different expression patterns of members of the *GrDEAD4-18-48* indicated that other *G. raimondii* helicase genes have been preserved by sub-functionalization.

The expressions of helicase genes were very diverse, even within one subgroup. For example, in the GrDExD/H group II, six genes (*GrDExD/H4*, *GrDExD/H30*, *GrDExD/H33*, *GrDExD/H38*, *GrDExD/H54*, and *GrDExD/H58*) were highly expressed in all three samples and clustered together. However, eight genes, including *GrDExD/H13* and *GrDExD/H45*, could only be detected in one to three organs, and were expressed at a low level in most organs. Nonetheless, given that as members in this group had a Q motif, they may be *GrDEAD* genes, which were assigned to the wrong subfamily owing to inadequate classification.

## 3. Discussion

RNA helicases play important roles in plant development and responses to stress. However, only a few RNA helicases have been identified in plants. Genome-wide analysis is the first step to elucidating the biological roles of members of the RNA helicase family members in certain plant species. The recent availability of genome sequences has enabled systematic investigation of this family of genes in *Arabidopsis*, rice, tomato, maize, and soybean. However, no RNA helicases have been characterized in cotton. This study involved a complete analysis of the RNA helicase gene family in the *G. raimondii* genome, including gene classification and the analysis of chromosomal locations, gene expansion, phylogenetic relationships, and structures of the genes, as well as their expression profiles at the fiber initiation stage and in mature leaves under normal growth conditions.

### 3.1. RNA Helicases in G. raimondii

We identified 161 RNA helicase genes in the diploid genome of the cotton species *G. raimondii* (Table 1). This is close to the estimated number of RNA helicase predicted from the analysis of the tomato (157), *Arabidopsis* (161), rice (149), maize (136), and soybean (213) genomes. The relatively large number of cotton RNA helicase genes identified likely reflects the detailed method we used to identify the RNA helicase genes. The presence of a large RNA helicase gene family in all of these species underscores that RNA helicases likely play important regulatory roles in various processes during plant growth and development. We classified these helicases into three subfamilies, which include the DEAD-box (51 genes), DEAH-box (52 genes), and DExD/H-box (58 genes) gene families (Table 1). Whereas, each of the three subfamilies of cotton RNA helicase genes consists of a similar number of genes, Xu *et al.* [6] have shown that the DEAD-box and DExD/H-box subfamily were larger than DEAH-box subfamily in *Arabidopsis*, rice, maize, and soybean. We also analyzed the predicted lengths, molecular weights, isoelectric points, and subcellular localizations of each putative helicase protein identified in the *G. raimondii* genome. We found that DEAD-box RNA helicases were distinct from DEAH-box and DExD/H-box RNA helicases. The apparently higher isoelectric points and smaller sizes of DEAD helicase proteins might be related to their relatively simple gene structures compared with other classes of RNA helicase. In addition, most of DEAD-box and DExD/H-box RNA helicase proteins were predicted to be located in the nucleus and cytoplasm while most DEAH-box RNA helicase proteins were predicted to reside in the nucleus (Table 1). Thus, we suggested DEAH-box RNA helicase proteins may mainly function in nuclear RNA processing. Linder *et al*. [34] have reported two DEAH-box RNA helicases, ESP3 and MUT6 which were located in nucleus perform different roles-RNA splicing and decay-in nuclear RNA processing. Several DEAD-box RNA helicase proteins and DExD/H-box RNA helicase proteins were predicted to be located in the chloroplast and mitochondria. Recently, there have been a few reports about RNA helicase proteins in chloroplast or mitochondria in plant. Asakura *et al*. [35] demonstrated that chloroplast RH3 DEAD-box RNA helicases in maize and *Arabidopsis* function in splicing of specific group II introns and affect chloroplast ribosome biogenesis. He *et al*. [36] demonstrated mitochondria ABO6 DExH-box RNA helicase in *Arabidopsis* involves in regulating the splicing of several genes of complex I.

### 3.2. Expansion of the RNA Helicase Gene Family in G. raimondii

Recent studies have shown that the *G. raimondii* genome has undergone at least two rounds of genome-wide duplication [25]. To detect possible relationships between RNA helicase genes and genome duplication events, we mapped 36 paralogous gene pairs (38.5%) of RNA helicase genes in *G. raimondii* (Figure 2, File S1). A similar percentage of paralogous pairs of RNA helicase genes was observed in *Arabidopsis*, rice, maize and soybean (35, 27, 25, and 62 pairs, respectively) [6]. These results suggest that the expansion of RNA helicase gene family is associated with whole-genome duplication events. However, the 38.5% value is much lower than that of the family of genes that encode NAC (NAM/ATAF/CUC) transcription factors (NAC-TFs) in *G. raimondii*. Of the 127 *G. raimondii* NAC-TF genes, 76.37% were within 307 identified syntenic blocks [37]. Given that segmental duplication events occur more often in the more slowly evolving gene families [38], the RNA helicase gene family may be evolving more rapidly than most other gene families in the cotton genome. In addition to the whole-genome duplication event, gene families can also arise through tandem amplification. For instance, in Chinese plum, tandem duplications played a key role in the expansion of the *AP2/ERF* family [39]. In *G. raimondii*, Shang *et al*. [37] also detected 20 tandem duplications associated with *NAC-TF* genes. However, only two gene pairs in our study showed evidence of having participated in tandem duplication. The mechanisms that supported the expansion of the RNA helicase gene family may be more complicated than is suggested by our classification; the specific mechanisms involved require further investigation. Marchat *et al*. [27] reported that the evolution of RNA helicase proteins involved gene fusion. Moreover, we speculate that given that the majority of RNA helicase gene family members play vital and diverse role in plants, there would be strong selection against variation in their copy numbers. The *DEAD* genes showed the greatest degree of duplication. Given the more simple and conserved gene structures and domains, as well as higher expression of members of this subfamily than those of others, we propose that DEAD helicase genes may evolve more slowly and may play more basic roles in plant growth and development than the members of other subfamilies of RNA helicases.

### 3.3. Phylogenetic Analysis and Gene Structural Organization

The full-length RNA helicase protein sequences in tomato and *G. raimondii* were aligned by MUSCLE and analyzed using the more accurate maximum likelihood (ML) method (File S2). Members of the DEAD-box, DEAH-box, and DExD/H-box subfamilies were further classified into four, six, and six subgroups, respectively (Figure 1), although Xu *et al*. [6] placed them into many more subclades following phylogenetic analysis of the RNA helicases of *Arabidopsis*, rice, maize, and soybean. Those workers classified the DEAD-box, DEAH-box, and DExD/H-box RNA helicase proteins from tomato into three, three, or five large subgroups, respectively. The diversity in the number and compositions of the subclades from different species indicate variation in the compositions of different RNA helicase gene families from different plant species. In addition, analysis of the exon-intron structures and sequences of the conserved domains can provide insights into the evolution of gene families. Our results showed that the numbers and locations of introns varied among subfamilies. Members of the DEAD-box subfamily had comparatively simple and conserved structural patterns. Genes from the GrDEAH-box and GrDExD/H-box subfamilies were less conserved and more diverse than those from the DEAD-box subfamily. It is noteworthy that genome-wide duplicated genes had the similar or same gene structures, whereas, tandem duplicated genes did not; this requires further analysis. Domain analysis indicated that an ATP-binding domain and a *C*-terminal domain were highly conserved in these putative RNA helicase proteins, whereas, more diversity was evident amongst the other domains present in each subfamily. The most characteristic feature of the DEAD-box family is the conserved Q motif [40]. We found Q motifs in all members of DEAD genes, except for *GrDEAD16*. In addition, three of the family members were found to have a WW domain. The WW domain has been implicated in mediating protein-protein interactions and linking cell signaling to the membrane cytoskeleton [41]. Whereas, DEAH genes lack a Q motif, most members of group II of DExD/H family have a Q motif. This might be attributed to the not-very-strict classification criteria used to distinguish between members of the DEAD and DExD/H subfamily. Many of the DEAH and DExD/H family genes were surrounded by defined folds, such as the zf-RING, dsRBD, and HAS domains, which may extend the length of the helicase. These regions influence or even define the function of a helicase [42]. The great diversity in these helicases may allow them to regulate many specific pathways in plants.

### 3.4. Expression Analysis Based on Transcriptome Sequencing Data

RNA helicases rearrange RNA secondary structure, potentially playing roles in any cellular process that involves RNA metabolism [3]. Of the 161 predicted genes in our study, 141 (87.6%) RNA helicase genes were expressed both in ovules and leaves. Xu *et al*. [6] reported that more than 80% RNA helicase genes in *Arabidopsis*, rice, and maize were expressed in at least one of the development stages and tissues tested. The high expression level of this RNA helicase gene family in all of these species further indicates that the RNA helicases may play important roles in various processes. The *GrDEAD* genes were more homogenous in terms of their level of expression and were expressed at higher levels both in ovules and leaves when compared with *GrDEAH* genes and *GrDExD/H* genes (Figure 5). The DEAD-box family member *GrDEAD7*, which is an ortholog of *Arabidopsis STRS1*, was highly expressed in 0-DPA ovules and mature leaves. The RNA helicases STRS1 and STRS2 have been shown to be involved in responses to various abiotic stresses [9]. *GrDEAD* genes may be more important in diverse cellular processes than *GrDEAH* genes and *GrDExD/H* genes. However, Xu *et al*. [6] have shown the DEAH-box RNA helicase genes higher proportion of the development stages and tissues in *Arabidopsis*, rice, and maize than DEAD-box RNA helicase genes and DExD/H-box RNA helicase genes. This phenomenon might be attributed to diversity between the different crops investigated or the diversity of the development stages and tissues examined. Moreover, tissue specificity of the expression of genes is commonly observed in plants. For example, cyclin dependent kinases-like proteins (CKL) of *Arabidopsis* show strong tissue specificity of expression, with *CKL12* being root specific, *CKL3* induced in stem tumors and callus, and *CKL6* showing strong expression only in leaf tissue [43]. Though *Arabidopsis* ISE2 has been shown to be involved in posttranscriptional gene silencing, and the absence of the ISE2 affects a critical factor required for correct plasmodesmata (PD) formation and function [16], the duration of PD closure was positively correlated with the final fiber length attained [44]. Thus, *GrDExD/H35* is not expressed at the initiation fiber stage.

## 4. Experimental Section

### 4.1. Identification of RNA Helicase Genes

The latest version (V2.0) of the *Gossypium raimondii* genome and protein sequences was downloaded from CottonGen [45]. To identify the members of the RNA helicase gene family in *Gossypium raimondii*, all known *Arabidopsis* helicase gene sequences and the identified tomato RNA helicase gene sequences [5] were used as queries to perform multiple database searches using BLASTP [46]. They were downloaded from The Arabidopsis Information Resource (TAIR) [47] and the plantGDB database [48], respectively. After selection of *G. raimondii* proteins with at least 50% identity with the query sequence, the candidate helicases proteins were aligned with each other to ensure that no gene was represented multiple times. All of the remaining protein sequences were examined using the domain analysis program PROSITE [49] with the default cutoff parameters. The three fields (length, molecular weight, and isoelectric point) of each *Gossypium raimondii* helicase protein were calculated using the online ExPasy program [50]. Subcellular localization was analyzed using the CELLO v2.5 server [51].

### 4.2. Phylogenetic Analysis

The full-length protein sequences of the helicase genes were aligned using the MUSCLE (v3.8.31) [30] program with the default settings. Phylogenetic trees were constructed by employing the maximum-likelihood (ML) method of the phyML (20120412) program [52] with the WAG (Whelan and Goldman) substitution model. Bootstrap values were calculated using the aLRT (approximate likelihood ratio test) model with the default cutoff parameters.

### 4.3. Chromosome Localization and Gene Duplications

Synteny analysis was conducted locally using a method similar to that developed for the Plant Genome Duplication Database [53]. Mcscan [54] was employed to identify homologous regions, and syntenic blocks were evaluated using Circos-0.64 [55]. Default parameters were used in all steps. Tandem duplication was characterized as multiple genes of one family located within the same or neighboring intergenic region [39].

### 4.4. Gene Structure and Domain Analysis

All of the putative protein sequences were analyzed using the domain analysis program PROSITE [56] at ExPASy [57]. Exon-intron structure information of these helicase genes was parsed from the GFF (Generic Feature Format) file downloaded along with the genomic data. Gene structures of the helicase genes were generated using the GSDS (Gene Structure Display Server) algorithm [58].

### 4.5. Gene Expression Analyses

The expression pattern of the helicase genes was analyzed using transcriptome sequencing data from mature leaves, 0-DPA ovules, and 3-DPA ovules of *G. raimondii*. These data were obtained from the NCBI Sequence Read Archive (SRA) [25]. The accession numbers were: SRX111367, SRX111365, and SRX111366, respectively. The search was performed using nucleotide signatures at least 20 nucleotide long. Reads mapping were performed by BWA (0.7.5a-r405) [59] with the default parameters except that the seed length was set to be 31. Sequenced reads that were mapped on these helicase sequences were converted to *RPKM* in order to estimate gene expression levels [60,61]. The formula used was:

$$RPKM=10^{6}C/\left(NL/10^{3}\right)$$

where *C* is the number of reads that were uniquely aligned to the transcript, *N* is the total number of reads that were uniquely aligned to all the transcripts in a specific sample and *L* is number of bases in the transcript.

## 5. Conclusions

Our study has reported a genome-wide analysis of the important RNA helicase gene family in *G. raimondii*. Based on their expression analysis, we hypothesize that GrDEAD37 and GrDExD/H35 might function during the fiber initiation stage. Only 38.5% of the putative RNA helicase genes were mapped to the previously identified syntenic blocks. The specific mechanisms used during the expansion of the RNA helicase family might be more complicated than suggested by our analysis, and require further investigation. Of the subfamilies of RNA helicases from *G. raimondii*, the GrDEADs have undergone the greatest degree of duplication and have the most conserved structural patterns and highest levels of expression, when measured at the level of transcript abundance. This suggests that the *GrDEAD* gene subfamily may evolve more slowly than the two other subfamilies, and that *GrDEAD* genes play a more important and basic role in cotton than *DEAH* or *DExD/H* genes. This study should provide a solid foundation for future functional studies and for guiding future experimental work on helicase genes in plants.

## Figures and Tables

**Table 1.** Characteristics of RNA helicases from *G. raimondii*. AA: Amino acid; *pI*: The theoretical isoelectric point of proteins; *M*_w_: The theoretical molecular weight of proteins.

Gene name	Gene identifier	Genomic position	Size (AA)	*M*_w_	*pI*	Subcellular localization
*GrDEAD1*	Gorai.001G008400.1	Chr01: 784950-787262	472	52.95	6.12	Cytoplasmic
*GrDEAD2*	Gorai.001G064600.1	Chr01: 6445636-6451393	604	68.98	8.93	Nuclear
*GrDEAD3*	Gorai.001G169800.1	Chr01: 24289833-24295275	499	57.13	8.66	Nuclear
*GrDEAD4*	Gorai.001G263500.1	Chr01: 53921207-53926399	655	70.98	9.19	Nuclear
*GrDEAD5*	Gorai.002G232200.1	Chr02: 59155342-59159290	620	67.94	8.4	Nuclear
*GrDEAD6*	Gorai.003G106600.1	Chr03: 32786814-32793828	622	71.26	8.96	Nuclear
*GrDEAD7*	Gorai.003G186400.1	Chr03: 45681420-45686317	564	62.89	8.97	Nuclear
*GrDEAD8*	Gorai.004G042200.1	Chr04: 3636522-3642658	490	56.2	8.84	Nuclear
*GrDEAD9*	Gorai.004G050000.1	Chr04: 4582045-4586206	776	85.09	5.72	Nuclear
*GrDEAD10*	Gorai.004G089100.1	Chr04: 11968779-11974240	450	49.77	6.53	Cytoplasmic
*GrDEAD11*	Gorai.004G157400.1	Chr04: 44565986-44571005	496	56.99	8.72	Nuclear
*GrDEAD12*	Gorai.004G277500.1	Chr04: 61046063-61050155	624	68.33	6.93	Chloroplast
*GrDEAD13*	Gorai.005G021200.1	Chr05: 1744957-1747872	683	78.2	8.94	Nuclear
*GrDEAD14*	Gorai.005G070600.1	Chr05: 7665522-7673295	1154	126.09	10.07	Nuclear
*GrDEAD15*	Gorai.005G084500.1	Chr05: 10403322-10409154	788	89.02	9.82	Nuclear
*GrDEAD16*	Gorai.005G118500.1	Chr05: 24460627-24464773	300	33.4	9.26	Cytoplasmic
*GrDEAD17*	Gorai.005G186000.1	Chr05: 54468800-54474652	580	63.66	7.68	Chloroplast
*GrDEAD18*	Gorai.006G031200.1	Chr06: 8129011-8134006	692	76.22	9.66	Nuclear
*GrDEAD19*	Gorai.006G095400.1	Chr06: 33433098-33436366	413	46.84	5.28	Cytoplasmic
*GrDEAD20*	Gorai.006G254000.1	Chr06: 49690002-49695784	814	91.89	9.16	Nuclear
*GrDEAD21*	Gorai.007G009400.1	Chr07: 733772-738313	697	75.73	8.99	Nuclear
*GrDEAD22*	Gorai.007G029000.1	Chr07: 1983857-1989372	349	39.22	8.92	Cytoplasmic
*GrDEAD23*	Gorai.007G047000.1	Chr07: 3268271-3272693	623	68.33	9.83	Nuclear
*GrDEAD24*	Gorai.007G097600.1	Chr07: 7210939-7217193	494	56.51	8.4	Nuclear
*GrDEAD25*	Gorai.007G107800.1	Chr07: 8134802-8139693	792	88.91	9.46	Nuclear
*GrDEAD26*	Gorai.007G226800.1	Chr07: 27332174-27337564	615	68.61	9.2	Nuclear
*GrDEAD27*	Gorai.007G304300.1	Chr07: 51812182-51818395	444	49.54	8.97	Cytoplasmic
*GrDEAD28*	Gorai.007G309800.1	Chr07: 52334743-52340317	820	90.34	8.33	Chloroplast
*GrDEAD29*	Gorai.007G362700.1	Chr07: 59429333-59434032	617	67.37	8.16	Nuclear
*GrDEAD30*	Gorai.008G282800.1	Chr08: 55902284-55906923	705	77.12	9.54	Nuclear
*GrDEAD31*	Gorai.008G245100.1	Chr08: 52991359-52996142	835	94.62	9.51	Nuclear
*GrDEAD32*	Gorai.009G043400.1	Chr09: 3162403-3166053	412	46.76	5.37	Cytoplasmic
*GrDEAD33*	Gorai.009G126600.1	Chr09: 9548083-9551194	587	65.9	6.7	Cytoplasmic
*GrDEAD34*	Gorai.009G140700.1	Chr09: 10637675-10642934	505	55.88	8.97	Cytoplasmic
*GrDEAD35*	Gorai.009G142800.1	Chr09: 10823077-10826736	413	47.01	5.29	Cytoplasmic
*GrDEAD36*	Gorai.009G193900.1	Chr09: 14896221-14903727	833	94.11	7.12	Nuclear
*GrDEAD37*	Gorai.009G410800.1	Chr09: 61968396-61972555	501	55.79	5.65	Cytoplasmic
*GrDEAD38*	Gorai.009G437700.1	Chr09: 68810677-68815318	752	84.97	8.96	Nuclear
*GrDEAD39*	Gorai.010G098500.1	Chr10: 17146619-17149615	413	46.77	5.37	Cytoplasmic
*GrDEAD40*	Gorai.010G134100.1	Chr10: 29846928-29852855	620	68.47	9.87	Mitochondrial
*GrDEAD41*	Gorai.011G063400.1	Chr11: 5308916-5312265	413	46.92	5.29	Cytoplasmic
*GrDEAD42*	Gorai.011G066000.1	Chr11: 5584414-5590538	505	55.72	9.04	Mitochondrial
*GrDEAD43*	Gorai.011G148000.1	Chr11: 24134100-24136116	661	76.36	8.62	Nuclear
*GrDEAD44*	Gorai.011G261800.1	Chr11: 59292567-59298642	1104	125.79	5.78	Nuclear
*GrDEAD45*	Gorai.011G288300.1	Chr11: 61987801-61991449	493	55.02	6.3	Cytoplasmic
*GrDEAD46*	Gorai.012G015300.1	Chr12: 1700644-1705443	598	68.17	9.48	Nuclear
*GrDEAD47*	Gorai.012G039900.1	Chr12: 4943976-4950340	643	69.16	9.51	Nuclear
*GrDEAD48*	Gorai.012G063000.1	Chr12: 8891463-8896643	688	75.16	9.8	Nuclear
*GrDEAD49*	Gorai.012G079600.1	Chr12: 12903543-12907932	593	66.23	9.11	Mitochondrial
*GrDEAD50*	Gorai.013G142600.1	Chr13: 38926934-38932273	444	49.59	8.97	Cytoplasmic
*GrDEAD51*	Gorai.013G247600.1	Chr13: 56620576-56625972	616	66.79	8.66	Nuclear
*GrDEAH1*	Gorai.001G141100.1	Chr01: 19085241-19093500	1328	148.92	1.97	Nuclear
*GrDEAH2*	Gorai.002G190800.1	Chr02: 51591530-51599433	979	109.02	9.01	Nuclear
*GrDEAH3*	Gorai.003G041600.1	Chr03: 5046943-5054254	1750	196.93	6.75	Nuclear
*GrDEAH4*	Gorai.004G081200.1	Chr04: 9735695-9741428	758	85.98	6.61	Nuclear
*GrDEAH5*	Gorai.004G164900.1	Chr04: 45891531-45901380	1615	182.54	7.34	Nuclear
*GrDEAH6*	Gorai.004G265100.1	Chr04: 60082397-60092032	711	80.98	6.59	Nuclear
*GrDEAH7*	Gorai.005G058400.1	Chr05: 6046005-6052692	1063	123.42	5.87	Nuclear
*GrDEAH8*	Gorai.005G157800.1	Chr05: 45129943-45137098	734	83.31	5.09	Nuclear
*GrDEAH9*	Gorai.005G161600.1	Chr05: 46720561-46734800	1028	114.74	5.99	Nuclear
*GrDEAH10*	Gorai.005G170100.1	Chr05: 49756283-49764261	700	78.71	7.56	Nuclear
*GrDEAH11*	Gorai.005G213400.1	Chr05: 59588117-59596596	1067	119.04	6.37	Nuclear
*GrDEAH12*	Gorai.006G000100.1	Chr06: 50440-58229	825	93.92	8.85	Cytoplasmic
*GrDEAH13*	Gorai.006G125500.1	Chr06: 37710924-37727582	1747	199.38	5.8	Nuclear
*GrDEAH14*	Gorai.006G135500.1	Chr06: 39140704-39151672	1536	174.79	8.3	Nuclear
*GrDEAH15*	Gorai.006G248800.1	Chr06: 49317977-49323365	1112	126.26	8.08	Nuclear
*GrDEAH16*	Gorai.006G264900.1	Chr06: 50379562-50386054	1065	123.47	5.93	Nuclear
*GrDEAH17*	Gorai.007G040200.1	Chr07: 2796320-2806189	1142	128.14	7.07	PlasmaMembrane
*GrDEAH18*	Gorai.007G136500.1	Chr07: 11144557-11150427	758	86.08	7.26	Nuclear
*GrDEAH19*	Gorai.007G190100.1	Chr07: 18534829-18540845	732	81.93	9.15	Nuclear
*GrDEAH20*	Gorai.007G273900.1	Chr07: 46703371-46719556	1484	168.81	5.48	Nuclear
*GrDEAH21*	Gorai.007G299200.1	Chr07: 51082328-51088099	882	99.76	8.49	Nuclear
*GrDEAH22*	Gorai.007G305400.1	Chr07: 51890852-51895676	611	68.07	8.18	Nuclear
*GrDEAH23*	Gorai.008G137800.1	Chr08: 38782533-38786182	725	82.66	7.22	Nuclear
*GrDEAH24*	Gorai.008G154400.1	Chr08: 41323487-41330255	885	100.52	7.91	Nuclear
*GrDEAH25*	Gorai.008G168800.1	Chr08: 44099126-44107869	731	82.91	5.38	Nuclear
*GrDEAH26*	Gorai.008G176500.1	Chr08: 45286639-45294517	718	80.5	8.88	Nuclear
*GrDEAH27*	Gorai.008G197200.1	Chr08: 48161550-48167878	1067	121.28	5.64	Nuclear
*GrDEAH28*	Gorai.009G117300.1	Chr09: 8628265-8633063	1189	135.53	6.56	Nuclear
*GrDEAH29*	Gorai.009G171000.1	Chr09: 13221402-13230043	1391	153.37	5.43	Nuclear
*GrDEAH30*	Gorai.009G186700.1	Chr09: 14354401-14365207	1234	137.83	8.29	Nuclear
*GrDEAH31*	Gorai.009G267400.1	Chr09: 22217004-22220722	1006	113.77	5.31	Nuclear
*GrDEAH32*	Gorai.010G093100.1	Chr10: 15093632-15104933	1675	187.55	6.05	Nuclear
*GrDEAH33*	Gorai.010G151100.1	Chr10: 40671899-40680492	1325	146.14	5.3	Nuclear
*GrDEAH34*	Gorai.010G228200.1	Chr10: 59997688-60013893	1021	115	8.53	Nuclear
*GrDEAH35*	Gorai.011G007600.1	Chr11: 569884-574434	1184	135.34	6.68	Nuclear
*GrDEAH36*	Gorai.011G032400.1	Chr11: 2409712-2417022	697	77.95	8.37	PlasmaMembrane
*GrDEAH37*	Gorai.011G075000.1	Chr11: 7156546-7161392	913	103.07	7.79	Nuclear
*GrDEAH38*	Gorai.011G111000.1	Chr11: 13326515-13334229	1760	197.96	7.62	Nuclear
*GrDEAH39*	Gorai.011G187000.1	Chr11: 44528408-44535161	1060	123.2	5.95	Nuclear
*GrDEAH40*	Gorai.011G206500.1	Chr11: 49795790-49814474	1767	199.65	7.03	Nuclear
*GrDEAH41*	Gorai.012G017400.1	Chr12: 1996523-2003747	1345	151.14	8.44	Nuclear
*GrDEAH42*	Gorai.012G136000.1	Chr12: 30913260-30920633	1038	114.76	6.6	Nuclear
*GrDEAH43*	Gorai.012G176400.1	Chr12: 34564794-34567329	658	74.12	6.1	Nuclear
*GrDEAH44*	Gorai.013G001200.1	Chr13: 68544-73180	863	96.21	8.9	Chloroplast
*GrDEAH45*	Gorai.013G038600.1	Chr13: 3074495-3083714	1203	135.39	7.84	Nuclear
*GrDEAH46*	Gorai.013G043100.1	Chr13: 3742541-3750413	971	107.92	6.2	Nuclear
*GrDEAH47*	Gorai.013G133100.1	Chr13: 35035921-35039609	990	112.45	5.64	Nuclear
*GrDEAH48*	Gorai.013G139300.1	Chr13: 37542094-37555083	1371	152.68	5.82	Nuclear
*GrDEAH49*	Gorai.013G141900.1	Chr13: 38687077-38695377	1232	139.18	6.13	Nuclear
*GrDEAH50*	Gorai.013G218600.1	Chr13: 53839220-53854330	2161	238.25	6.56	Nuclear
*GrDEAH51*	Gorai.013G252300.1	Chr13: 56979166-56992254	1773	202.64	5.77	Nuclear
*GrDEAH52*	Gorai.013G258600.1	Chr13: 57397754-57406240	1037	115.47	7.45	Nuclear
*GrDExD/H1*	Gorai.001G036900.1	Chr01: 3403877-3420237	1455	165.33	5.25	Nuclear
*GrDExD/H2*	Gorai.001G150400.1	Chr01: 20836533-20846178	1950	218.72	5.96	Nuclear
*GrDExD/H3*	Gorai.001G168700.1	Chr01: 24030814-24041949	2238	251.24	9	Nuclear
*GrDExD/H4*	Gorai.002G016100.1	Chr02: 1050235-1053354	405	45.9	5.99	Cytoplasmic
*GrDExD/H5*	Gorai.002G143000.1	Chr02: 25828154-25839065	1393	158.07	6.64	PlasmaMembrane
*GrDExD/H6*	Gorai.002G216000.1	Chr02: 56556123-56573745	1470	163.86	6.4	Nuclear
*GrDExD/H7*	Gorai.003G100200.1	Chr03: 30861094-30875047	1287	144.52	6.5	Nuclear
*GrDExD/H8*	Gorai.003G125100.1	Chr03: 37290357-37302757	1340	149.47	5.67	Cytoplasmic
*GrDExD/H9*	Gorai.003G156900.1	Chr03: 42640922-42647039	796	90.08	8.15	Nuclear
*GrDExD/H10*	Gorai.004G209000.1	Chr04: 54157901-54160653	548	59.42	9.63	Mitochondrial
*GrDExD/H11*	Gorai.004G221400.1	Chr04: 55605032-55610980	1225	137.54	6.4	Nuclear
*GrDExD/H12*	Gorai.004G264200.1	Chr04: 59938105-59956863	2042	231.37	5.47	Nuclear
*GrDExD/H13*	Gorai.004G292500.1	Chr04: 62043821-62048421	428	48.46	5.4	Cytoplasmic
*GrDExD/H14*	Gorai.005G137100.1	Chr05: 35497500-35505536	926	104.62	8.77	Nuclear
*GrDExD/H15*	Gorai.005G170000.1	Chr05: 49691064-49693215	401	45.32	9.41	Mitochondrial
*GrDExD/H16*	Gorai.005G194600.1	Chr05: 56476340-56494984	1217	137.04	7.12	Nuclear
*GrDExD/H17*	Gorai.006G005000.1	Chr06: 967752-981201	1063	120.14	5.98	Nuclear
*GrDExD/H18*	Gorai.006G017900.1	Chr06: 4179363-4184250	851	97.23	6.43	Nuclear
*GrDExD/H19*	Gorai.006G241400.1	Chr06: 48762774-48767537	563	63.2	8.72	Mitochondrial
*GrDExD/H20*	Gorai.006G257000.1	Chr06: 49880967-49896698	2586	283.31	6.46	Nuclear
*GrDExD/H21*	Gorai.007G006300.1	Chr07: 523949-533327	1196	14.44	8.96	Nuclear
*GrDExD/H22*	Gorai.007G023800.1	Chr07: 1694751-1699635	770	86.86	8.08	Nuclear
*GrDExD/H23*	Gorai.007G100800.1	Chr07: 7468905-7480135	2258	253.19	8.82	Nuclear
*GrDExD/H24*	Gorai.007G374300.1	Chr07: 60633068-60637091	532	59.72	9.39	Nuclear
*GrDExD/H25*	Gorai.008G031000.1	Chr08: 3714524-3722015	930	105.31	6.29	Nuclear
*GrDExD/H26*	Gorai.008G049300.1	Chr08: 6883759-6898577	2377	263.01	7.2	Nuclear
*GrDExD/H27*	Gorai.008G068400.1	Chr08: 11250164-11254784	748	85.76	5.76	Nuclear
*GrDExD/H28*	Gorai.008G121100.1	Chr08: 35933836-35944775	2250	252.23	8.95	Nuclear
*GrDExD/H29*	Gorai.008G145400.1	Chr08: 39788075-39795065	1017	115.15	6.02	PlasmaMembrane
*GrDExD/H30*	Gorai.008G149500.1	Chr08: 40485456-40488718	408	46.16	5.98	Nuclear
*GrDExD/H31*	Gorai.008G198400.1	Chr08: 48292290-48321142	2090	236.59	6.08	Nuclear
*GrDExD/H32*	Gorai.009G053300.1	Chr09: 3872981-3886515	1654	186.18	6.7	Nuclear
*GrDExD/H33*	Gorai.009G057600.1	Chr09: 4145298-4149685	428	48.49	5.41	Cytoplasmic
*GrDExD/H34*	Gorai.009G153000.1	Chr09: 11690907-11694880	514	57.39	9.54	Nuclear
*GrDExD/H35*	Gorai.009G345500.1	Chr09: 41630420-41637165	1179	133.41	5.3	Nuclear
*GrDExD/H36*	Gorai.009G374900.1	Chr09: 50970390-50979491	1395	158.66	6.56	PlasmaMembrane
*GrDExD/H37*	Gorai.009G393800.1	Chr09: 54260282-54269764	1035	115.37	8.81	Nuclear
*GrDExD/H38*	Gorai.010G114300.1	Chr10: 22016499-22022004	428	48.44	5.41	Cytoplasmic
*GrDExD/H39*	Gorai.010G134200.1	Chr10: 29886669-29889520	409	45.73	9.2	Nuclear
*GrDExD/H40*	Gorai.010G135100.1	Chr10: 30499026-30514000	688	77	8.43	Cytoplasmic
*GrDExD/H41*	Gorai.010G175700.1	Chr10: 51517626-51529268	991	112.56	6.05	Nuclear
*GrDExD/H42*	Gorai.010G194700.1	Chr10: 55411136-55418053	1268	146.35	8.06	Nuclear
*GrDExD/H43*	Gorai.010G244100.1	Chr10: 61318853-61332614	1462	166.45	5.32	Nuclear
*GrDExD/H44*	Gorai.011G082500.1	Chr11: 8316574-8321873	427	48.38	5.4	Cytoplasmic
*GrDExD/H45*	Gorai.011G088700.1	Chr11: 9302151-9305910	535	58.4	8.55	Nuclear
*GrDExD/H46*	Gorai.011G165900.1	Chr11: 32740783-32745005	481	54.19	9	Nuclear
*GrDExD/H47*	Gorai.011G166100.1	Chr11: 32787373-32798664	1114	128.03	6.23	Nuclear
*GrDExD/H48*	Gorai.011G185100.1	Chr11: 43975253-43992851	3361	365.5	5.33	Nuclear
*GrDExD/H49*	Gorai.011G217300.1	Chr11: 52330942-52338804	2177	247.23	5.53	Cytoplasmic
*GrDExD/H50*	Gorai.012G023100.1	Chr12: 2830126-2846019	1138	128.76	8.88	Nuclear
*GrDExD/H51*	Gorai.012G039300.1	Chr12: 4882407-4892116	1221	134.91	6.11	Nuclear
*GrDExD/H52*	Gorai.012G063400.1	Chr12: 8945198-8952950	1210	135.5	8.5	Nuclear
*GrDExD/H53*	Gorai.013G034700.1	Chr13: 2677455-2684701	982	111.62	7.1	Nuclear
*GrDExD/H54*	Gorai.013G070700.1	Chr13: 8189231-8192867	406	45.98	5.98	Nuclear
*GrDExD/H55*	Gorai.013G146500.1	Chr13: 40255533-40278859	1256	139.06	7.25	Nuclear
*GrDExD/H56*	Gorai.013G197700.1	Chr13: 50405230-50425237	2054	227.78	5.83	Nuclear
*GrDExD/H57*	Gorai.013G211300.1	Chr13: 52317728-52328526	990	111.41	6.26	Cytoplasmic
*GrDExD/H58*	Gorai.013G215200.1	Chr13: 53550658-53555657	428	48.44	5.4	Cytoplasmic
